# Risodontropy

**Published:** 1853-04

**Authors:** John S. Clark


### Editorial.

### RISODONTRYPY.

The reported cases of Prof. Cone of exposed nerves treated by this operation and which was crowded out of our last, we give in this number. We have no doubt they will be read with interest. We have been successfully resorting to this treatment since our last publication, and have one or two suggestions which we think of service. The bow drill, is not the one we have found best adapted. We have a very superior screw stock drill which we supposed would be even better than the bow drill, but both we have found objectionable, and now use a drill or drills, made from our steel handled excavators after the points have been broken off. And we prefer them not too much spear-pointed, because when of this shape they slip too suddenly into the nerve cavity, thus exposing the point to be broken off and left in, for they will generally have to remain. This is the difficulty with the bow drill, for we cannot so accurately feel if the point of the drill is approaching the nerve. But with the steel handled drill worked with the fingers, we can tell, and by care prevent an accident of this kind.

In healthy exposed nerves, so far, we have been successful, and have in some few cases treated deceased nerves by the same operation, having first by other means allayed inflammation. In a superior anterior molar which had been painful for several days, without swelling, after subduing the general inflammation, we drilled under the margin of the gum pointing toward the pulp cavity, and after wounding this, we plugged the tooth in its posterior approximal surface. Some soreness and pain from extremes of heat and cold followed, which was subdued by the use of Tincture of Arnica rubbed on the gum around the tooth. After about six weeks, pain set in again, and we introduced a probe and found the pulp alive. The wounding of the nerve in probing the tooth relieved the pain and the tooth still remains. The ultimate result is doubtful, most likely, however, the tooth will be lost.

At the Dental College, one tooth, treated in this manner by one of the class was afterwards extracted.

There is one condition of the teeth where we believe this operation will prove of great service. We allude to that condition where the nerve is nearly exposed by the wearing down of the teeth. Some persons suffer a great deal from this cause and loose many of their teeth when perfectly sound. It certainly would be better practice here to cut the nerve above the neck of the tooth, than to expose and destroy then plug, from our short experience in this practice, we have no doubt but that we shall be enabled to save many teeth which formerly gave us much trouble. The operation of Risodontrypy; Dr. Clark of N. O. informs us he has been performing since 1844. We give an extract from his letter which fully explains his views. &c.

"I am not going to set up a claim, to priority in the operation now named 'Risodontrypy.' But I have performed that operation many times, I should think one hundred, dating as far back as 1844, but it was for a different purpose, and under different indications.

"I have a student with me now who has been with me nearly eight years, and when he read the first article on that subject he said he had seen me perform that operation at least 20 times. The way I came to do it was, that I saw a patient of Dr. Hale of St. Louis, for whom the Dr. had operated, and drilled into the nerve channel for the escape of pus. I followed suit, but carried it farther. After filling a tooth by caping the nerve on the first exhibition of pain tending as I suppose to suppuration, I at once drilled into the nerve channel, but sliped the drill up above the gum for the purpose of having the gum act as a valve to prevent ingress of air or foreign substances.

"I have not time to write in extenso as to cases, but I will give in a few words, as I suppose, results. It is true, many of those teeth when drilled before suppuration had commenced seemed to change but little, but I abandoned it in '48 supposing it to be mal-practice, and let no tooth go out of my hands (with exposed nerves,) without attempting extirpation and fang filling. My belief has been and even now is so far as my experience goes that the pulp (which I know seems to retain some of its functions) becomes a sort of fungus retaining blood in excess and a sort of vitality, but the vitality *of a fungus only.* I hope I am wrong. and day before yesterday, I performed the operation according to the plan proposed, begging pardon of the discoverer for not using the drill *Bow* except the digital extremities of a (beaux) bow of antiquity. Hardly a day has passed over my head for three months, (except Sunday,) that I have not filled a fang or fangs, I have usually a half a dozen under treatment and more at present writing. Have filled an upper molar and Bis. to-day, fangs and crown. As to honors in the new operation, I say, God speed to the man who can advance the profession one step towards 'that good time coming.' But I must close.

Pardon this long, hurried letter, and Believe me,

Yours Fraternally,

JOHN S. CLARK.

Drilling into the nerve cavity for the escape of matter, is, we are fully aware, an old operation. But Dr. Hullihen introduces this operation for a different purpose and under different circumstances, the object, it is true, may be the same as that of M. L. Fatori's, but only so far as severing the nerve is concerned. The latter instead of cutting the nerve at the ear ; cuts it at the extremity of the fang, and does this as a remedy for the tooth ache.

